# Ultrasound Super‐Resolution Imaging of Neonatal Cerebral Vascular Reorganization

**DOI:** 10.1002/advs.202415235

**Published:** 2025-02-03

**Authors:** Simone Schwarz, Louise Denis, Emmanuel Nedoschill, Adrian Buehler, Vera Danko, Alina C. Hilger, Francisco Brevis Nuñez, Nikola R. Dürr, Martin Schlunz‐Hendann, Friedhelm Brassel, Ursula Felderhoff‐Müser, Heiko Reutter, Joachim Woelfle, Jörg Jüngert, Christian Dohna‐Schwake, Nora Bruns, Adrian P. Regensburger, Olivier Couture, Henriette Mandelbaum, Ferdinand Knieling

**Affiliations:** ^1^ Department of Neonatology and Pediatric Intensive Care Medicine Sana Clinics Duisburg Zu den Rehwiesen 9 47055 Duisburg Germany; ^2^ Department of Pediatrics I University Hospital Essen University of Duisburg‐Essen Hufelandstraße 55 45147 Essen Germany; ^3^ Centre for Translational Neuro‐ and Behavioral Sciences University Hospital Essen University of Duisburg‐Essen Hufelandstraße 55 45147 Essen Germany; ^4^ Laboratoire d'Imagerie Biomédicale Sorbonne Université CNRS INSERM 15 Rue de l'Ecole de Médecine 75006 Paris France; ^5^ Department of Pediatrics and Adolescent Medicine University Hospital Erlangen Loschgestraße 15 91054 Erlangen Germany; ^6^ Clinic for Radiology and Neuroradiology Sana Clinics Duisburg Zu den Rehwiesen 9 47055 Duisburg Germany

**Keywords:** CEUS, microbubbles, super‐resolution imaging, ultrasound localization microscopy, ultrasound, Vein of Galen malformation

## Abstract

During the first days of neonatal growth, the central nervous system (CNS) develops self‐regulatory mechanisms to ensure constant cerebral perfusion. However, this vascular neogenesis takes place at a microscopic scale that cannot be observed with current clinical imaging techniques. Ultrasound localization microscopy (ULM) allows us to observe micro‐vessels of the order of a few microns at depths of several centimeters. This can be done using conventional clinical ultrasound scanners and contrast sequences (CEUS). In this study, ULM is used to observe the human microvasculature in neonatal patients undergoing treatment for life‐threatening malformations forming direct connections between the cerebral arterial and venous systems. It is observed that neuroendovascular treatment of neonatal arteriovenous malformations causes remodeling and reorganization of the cerebral vasculature by also activating corticomedullary vascular connections. ULM enables us to follow microvascular changes in human neonates with high spatio‐temporal resolution. ULM may provide a novel clinical translatable tool, particularly including cerebral imaging in very young patients.

## Graphical abstract

The scale of neonatal microvasculature is currently beyond the resolution of clinical imaging techniques. In this study, ultrasound localization microscopy is used to observe the microvasculature in neonates undergoing treatments for life‐threatening vascular malformations. ULM enables the imaging and quantification of microvascular changes with high spatio‐temporal resolution providing a novel clinical tool for cerebral imaging in very young patients.

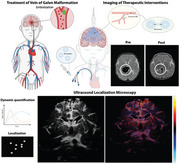

## 1 Introduction

The overall integrity of the brain is dependent on the continuous supply of nutrients and oxygen through a highly interconnected vascular system, which provides ≈20% of the cardiac blood flow to the adult brain.^[^ ^1^ ^]^ Until now, capturing in vivo dynamical adaptation remains challenging with conventional imaging methods. Understanding the contribution of microcirculation, in conditions such as cerebral small vessel disease, has facilitated the development of advanced neuroimaging techniques.^[^ ^2^ ^]^ In this regard, advanced computed tomography (CT) or magnetic resonance imaging (MRI) are used for the observation of cerebral perfusion^[^ ^3^ ^]^ but are not routinely implemented in the clinical workup – especially not for newborns and children. In newborns, high‐resolution, radiation‐free ultrasound (US) through the open fontanelle is commonly used to assess microvascular structures and pathologies such as preterm or hypoxic injury.^[^ ^4^ ^]^ It is possible that even the smallest changes to the newborn brain can have long‐term effects on development, so that the ability to assess macrovascular alterations may have a decisive influence on clinical decision making.^[^ ^5^ ^]^

Recently, contrast‐enhanced ultrasound (CEUS), a technique using intravascular echogenic gas‐filled microbubbles has been employed to study the kinetics of microbubble flow in cerebral vasculature of neonates.^[^ ^6^ ^]^ These microbubbles circulate strictly intravascularly,^[^ ^7^ ^]^ are independent from liver and kidney clearance^[^ ^7^, ^8^ ^]^ and therefore provide excellent applicability in clinical vascular imaging.^[^ ^9^ ^]^ Moreover, the tracking of individual microbubbles allows for super‐resolution imaging, so called ultrasound localization microscopy (ULM)^[^ ^9^, ^10^ ^]^ offering insights into adaptations on a microvascular scale.^[^ ^9^ ^]^ Given these capabilities, we investigated the feasibility to monitor macro‐ and microstructural circulatory changes following therapeutic interventions on the brain of newborns. We study subjects suffering from Vein of Galen (aneurysmal) malformation (VGAM) – a rare disease that is caused by direct connections between the intracranial arterial and venous systems.^[^ ^11^ ^]^ Without treatment, this condition is fatal because of multiple organ failure or complications from hydrocephalus in the first year of life.^[^ ^12^ ^]^

Here, we demonstrate that functional and ultrastructural US measurements can be used to visualize the microvascular architecture and its complex flow changes during endovascular therapy. Using readily available clinical hardware, it is possible to monitor the dynamic adaptations of microcirculation in different brain regions providing novel information on disease pathology and treatment outcome.

## 2 Results

### 2.1 Clinical Course of Vein of Galen Malformation Patients

To study the therapeutic effects of neurovascular, catheter‐based approaches on cerebral vasculature, we included *n* = 7 consecutive neonates without exclusion diagnosed with VGAM at a median gestational age of 37 1/7 (range 34 6/7–40 1/7), a median admission weight of 3000 g (range 2450–4600 g) and a median postnatal age of 1 day (range 1–2 days) (Figure S1A, Table S1, Supporting Information). The subjects were clinically evaluated for the extent/type of malformation and received standard clinical imaging, including US and MRI, as a prerequisite for further investigations (Figure S1B, Table S2, Supporting Information). All neonates received endovascular therapy at a median postnatal age of 2 days (range 2–9 days). The exact timing was individually determined according to the clinical course together with logistic capacities and availability of the entire treatment team. Embolization was performed using a combined therapy with arterial and venous accesses for precise closure of high‐flow arteriovenous fistulas exactly at the shunting point schematically shown in Figure S1C,D (Supporting Information). 3/7 infants (43%) experienced serious adverse events during or after embolization. One neonate developed a hemorrhagic infarction of the right thalamus and corpus callosum with extension into the peritrigonal medullary bed, subsequent with intraventricular hemorrhage and posthemorrhagic hydrocephalus one week after the first embolization. After discharge, a ventriculoperitoneal shunt was required. One neonate suffered temporooccipital hemorrhage with mass effect and compression of the midbrain and brainstem. Neurosurgical decompression of the hemorrhage could not prevent irreversible brain damage, and the neonate died on the 5th day of life after withdrawal of life support. One neonate exhibited a hemorrhage in the posterior fossa, resulting in damage to the brain stem, after the third embolization procedure. This patient deceased on day 26th of life under palliative care. Interventional complications are usually caused by direct intraprocedural arterial perforation or by complex changes in cerebral hemodynamics due to occlusion of the pathological arteriovenous connections.^[^ ^13^ ^]^ 5/7 (72%) patients were discharged alive. To investigate the effects of the procedure, transfontanellar CEUS (T‐CEUS) imaging was performed with an injection of ultrasound contrast agent (Sonovue, Bracco, Italy) (Figure S1E, Supporting Information). For the final analysis, a total of *n* = 20 investigations at the three time points were included (T1–T3 in six patients, T1–T2 in the patient who dieted on the 5th day of life).

### 2.2 Cerebral Perfusion Dynamics During Therapy

**Figure 1:**
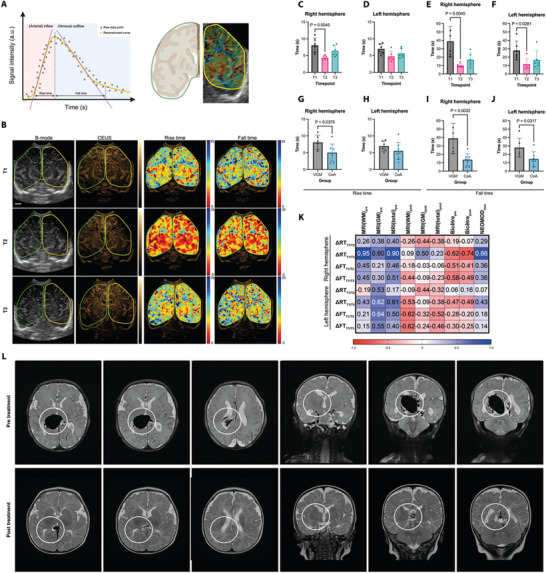
Perfusion dynamics of the cerebral vasculature during therapy. A) Schematic illustration of dynamic flow parameter measurements in both hemispheres. Rise (RT) and fall time (FT) can be retrieved from the time‐intensity curve of the overall microbubble signal. B) Representative ultrasound (US), contrast‐enhanced ultrasound (CEUS), and color‐coded maps of rise (RT) and fall time (FT) (left to right) during therapy (T1–T3, upper to lower rows) of a single patient. More red color‐coding indicates an increase in rise time (faster arterial inflow). Identical measurements were performed in *n* = 7 consecutive patients with Vein of Galen malformation. C) RT measurement of the right and D) left hemisphere for T1–T3. E) FT measurement of the right and F) left hemisphere for T1‐T3. Parameters in relation to baseline were tested with the Kruskal‐Wallis test and Dunn's post‐test. G−J) Comparison of RT and FT of the first time point in both hemispheres to *n* = 8 patients with no cerebral pathology. Significance tested with unpaired, non‐parametric *t*‐tests. K) Correlation matrix using Spearman's correlations coefficient comparing reduction in RT (ΔRT) and FT (ΔFT) with white (MRI(WM)_pre_), grey matter (MRI(GM)_pre_) and total magnetic resonance imaging scores (MRI(total)_pre_) before and after (MRI(WM)_post_, MRI(GM)_post,_ MRI(total)_post_) intervention (The WM score consisted of four grades: normal is 5–6 points, mildly abnormal is 7–9, moderately abnormal is 10–12, and severely abnormal 13–15. Pre‐ and post‐treatment grey matter abnormalities were assessed using a three‐point scale in three regions. The total GM score was calculated by summing up the points from each region and graded into normal (3–5 points) and abnormal (6–9). Using GM and WM subscores, a total score was calculated. A total score of 19–24 points was considered as severe brain injury), Bicêtre neonatal evaluation score before (Bicêtre_pre_) and after (Bicêtre_post_) (Bicêtre neonatal evaluation score evaluates five objective clinical parameters: cardiac function, respiratory function, cerebral function, renal function, and hepatic function, resulting in a score between 0–21 points, lower scores indicate higher disease severity)^[^ ^11b^ ^]^ and maximum neonatal multiple organ dysfunction scores (NEOMOD_max_) (The NEOMOD assessment scores eight organ systems: central nervous, hemocoagulation balance, respiratory, gastrointestinal, cardiovascular, renal, acid‐base balance, and microvascular systems, with a score between 0 and 16 points, higher scores indicate higher disease severity).^[^ ^14a^ ^]^ L) Magnetic resonance imaging of a single patient pre and post‐treatment. White circles represent the area of the malformation. Created with BioRender.com.

First, we quantified the time‐intensity curves as a measure of the flow of microbubbles in the vessels. **Figure** **1**A shows the methodology to derive dynamic flow parameters within a respective region of interest of the brain. To describe the changes in perfusion, we used measures for arterial inflow (rise time, RT) and venous outflow (fall time, FT). We observed a visible change in dynamic perfusion parameters from pre‐treatment (T1) to within 24 h post‐treatment (T2, Figure 1B). Especially in the right hemisphere a decrease in rise time (RT) from T1 (8.0 ± 2.2 s) to T2 (4.4 ± 0.6 s, *p* = 0.0045) was seen, which was unchanged in the left hemisphere (Figure 1C,D). Furthermore, the fall time (FT) decreased similarly in the right hemisphere from T1 (38.9 ± 17.9 s) to T2 (10.0 ± 2.3 s, *p* = 0.0045) and the left hemisphere (28.0 ± 11.3 s vs 11.7 ± 6.4 s, *p* = 0.0281) (Figure 1E,F). To a lesser extent, these effects were still visible approximately one week after therapy (T3). By comparing these RT and FT measurements to a historic cohort of *n* = 8 patients with coarctation of the aorta (CoA),^[^ ^6c^ ^]^ which should not have a cerebral involvement of their disease, we found that both a longer RT and FT prior to therapy was a hallmark of VGAM patients (Figure 1G–J). Moreover, treatment interventions result in a decrease in RT and FT that more closely resembles physiologic values seen in these other unaffected patients.

### 2.3 Imaging as Indicator for Treatment Effects After Endovascular Therapy

Next, we correlated the decrease in RT and FT between timepoints T1 versus T2 (ΔRT_T1/T2_ and ΔFT _T1/T2_) and T1 versus T3 (ΔRT_T1/T3_ and ΔFT_T1/T3_), as a measure for individual response, with the individual clinical outcome (Bicêtre score,^[^ ^11b^ ^]^ NEOMOD score).^[^ ^14^ ^]^ ΔRT_T1/T3_ on the right hemisphere demonstrated negative correlations with pre (*R*_s_ = −0.62, *p* = 0.152) and post Bicêtre scores (*R_s_* = −0.74, *p* = 0.071) and positive correlation with maximum Neonatal Multiple Organ Dysfunction Score (NEOMODmax, *R*_s_ = 0.86, *p* = 0.024). Furthermore, we found positive correlations with pre‐interventional MRI scores (e.g., MRI(total)_pre_, *R_s_* = 0.90, *p* = 0.013) for brain damage (Figure 1K).^[^ ^15^ ^]^ Given these correlations, this indicates a (therapeutic) effect of embolization, which was supposedly larger in more severely affected patients. Figure 1L shows the MRI findings of a single patient pre and post‐treatment.

### 2.4 Treatment Effects in Different Brain Regions

**Figure 2:**
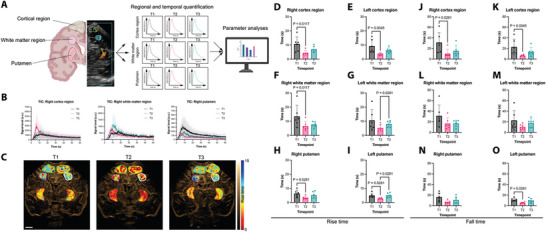
Macrostructural adaptions of the cerebral vasculature during therapy. A) Schematic illustration of dynamic flow parameter measurements in different cerebral regions during all time points (T1–T3). B) Representative mean time‐intensity curves with standard deviation (bright area) during therapy (T1–T3) in *n* = 7 patients. C) Representative color‐coded maps of rise time (RT) during therapy (T1–T3) of a single patient. Bar represents 1cm. D) RT measurement of the right and E) left cortex, F) right, and (G) left white matter, H) right and I) left putamen. J − O) Similar measurements on the left hemispherical regions. Parameters in relation to baseline were tested with the Kruskal‐Wallis test and Dunn's post‐test. Bars represent SD. Created with BioRender.com.

To closely decipher spatial reorganization after neurovascular interventions, we separately analyzed different anatomical regions. Brain regions of both hemispheres were divided in the cortical region (including grey matter and subcortical white matter), white matter region, and putamen (**Figure** **2**A). We found that the generated time‐intensity curves from each region underwent marked changes during therapy (Figure 2B) and mapped the respective flow parameters (Figure 2C). Like our measurement of the whole hemispheres, we found a decrease in RT. This indicates a steeper arterial blood flow, more in the cortical area than in other regions. (Figure 2D–I). At the same time, the venous congestion seems to improve as the venous outflow is much faster, which is indicated by the FT (Figure 2J–O). Vascular effects are best visualized directly after the acute intervention in the cortical region (T2) and are only to a small extent still visible in the course after one week (T3), possibly reflecting post‐interventional hyperperfusion.^[^ ^13^, ^16^ ^]^

### 2.5 Microvascular Imaging of Blood Flow Dynamics During Therapy

**Figure 3:**
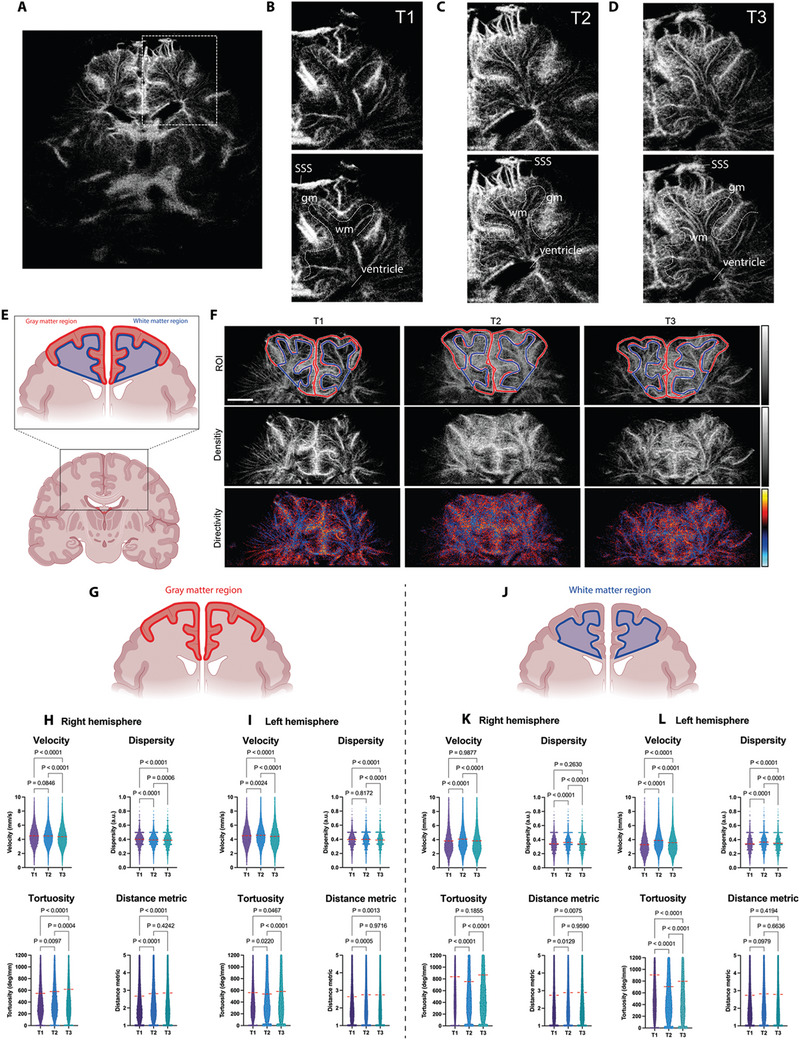
Identification of cortical layers to measure microvascular blood flow dynamics during therapy. A) The dashed box represents an example of the selection of the neonatal cortex to be analyzed. B) The upper row shows the magnified region and the lower outline of the grey (gm) and white matter (wm) regions, which are clearly separated by different vascular localizations. SSS = superior sagittal sinus. The following images show the identical section for time points T2 C) and T3 D). Schematic illustration and representative images for analyzed grey and white matter regions E). The upper row outlines the grey (red) and white matter (blue) regions, the middle row a density map, and the lower row a directivity map. Bar represents 1 cm F). Outline of the grey matter region G). Microbubble flow velocities (T1: *n* = 15 174, T2: *n* = 17 710, T3: *n* = 19 596), dispersity (T1: *n* = 15 156, T2: *n* = 17 711, T3: *n* = 19 515), tortuosity (T1: *n* = 15 585, T2: *n* = 18 209, T3: *n* = 20 027) and distance metric (T1: *n* = 15 587, T2: *n* = 18 210, T3: *n* = 20 027) for right grey matter region and all time points H). Microbubble flow velocities (T1: *n* = 14 841, T2: *n* = 20 149, T3: *n* = 19 685), dispersity (T1: *n* = 14 597, T2: *n* = 19 698, T3: *n* = 19 192), tortuosity (T1: *n* = 15 116, T2: *n* = 20 569, T3: *n* = 20 152) and distance metric (T1: *n* = 15 115, T2: *n* = 20 571, T3: *n* = 20 153) for left grey matter region and all time points I). Outline of the white matter region J). Microbubble flow velocities (T1: *n* = 14 841, T2: *n* = 19 814, T3: *n* = 17 600), dispersity (T1: *n* = 10 146, T2: *n* = 12 969, T3: *n* = 11 421), tortuosity (T1: *n* = 10 929, T2: *n* = 13 676, T3: *n* = 11 941) and distance metric (T1: *n* = 10 931, T2: *n* = 13 676, T3: *n* = 11 944) for right white matter region and all time points K). Microbubble flow velocities (T1: *n* = 10 167, T2: *n* = 14 440, T3: *n* = 13 918), dispersity (T1: *n* = 9940, T2: *n* = 14 305, T3: *n* = 13 333), tortuosity (T1: *n* = 10 514, T2: *n* = 14 825, T3: *n* = 14 257) and distance metric for left white matter region and all time points L). The Red dashed line indicates the mean. Parameters tested with one‐way ANOVA with Tukey post‐test. Created with BioRender.com.

After dynamic, quantitative T‐CEUS indicated a direct post‐interventional effect on blood flow close to the cortex, we wanted to better resolve the anatomy within this region in the next step. Subsequently, we performed a reconstruction of vascular mapping using ultrasound localization microscopy (ULM). By tracking the movement of individual microbubbles, we derived density and directivity maps for all patients at the three‐time points (T1–T3, total of *n* 20 scans). With the use of ULM, we were able to clearly outline the different cortical zones, which is not feasible with standard ultrasound (**Figure** **3**A–C). For the exact anatomical analyses, we segmented the visualized cortical grey and subcortical white matter regions for every time point. Using more than 15.000 individual microbubble velocities per time point, we found a steady decrease in flow velocity in the right hemisphere grey matter when comparing baseline of 4.5 ± 1.5 (T1) to 4.5 ± 1.4 mm s^−1^ (*p* = 0.0846) 24 h after the intervention (T2) to 4.4 ± 1.6 mm s^−1^ (*p* < 0.0001) after one week (T3) (Figure 3E–H). Furthermore, dispersity decreased, and tortuosity and distance metrics increased. Taken together, this might suggest a complex persistent cortical reorganization taking place after endovascular intervention. Similar results were found in the left hemisphere grey matter (Figure 3I). In contrast to the grey matter regions, the therapy had a different effect on the segmented white matter regions (Figure 3J). For example, the flow velocity in the right white matter was only temporarily increased from 3.8 ± 2.0 mm s^−1^ at baseline (T1) to 4.1 ± 1.9 mm s^−1^ (*p* < 0.0001) 24 h after therapy (T2) back to 3.8 ± 2.0 mm s^−1^ (*p* = 0.9877) after one week (T3) (Figure 3K). However, we found consistency between right and left white matter, especially concerning dispersity, tortuosity, and distance metric. More interestingly, trends in grey and white matter regions follow independent logics. As a result of the intervention, we observed an increase in the flow velocity in the white matter while the flow velocity in the grey matter decreased (Figure 3L). In the white matter, the detection of longer venous vessels presumably predominates over the more tortuous arteries and veins of the cortical grey matter. In this regard, the microvascular changes indicate improved venous outflow in the white matter. In contrast, we interpret the changes in the cortical grey matter as evidence of improved perfusion after occlusion of arteriovenous shunts, mainly due to an increase in blood flow in the small microvessels showing an increased vessel branching with an overall loss of velocity.

**Figure 4:**
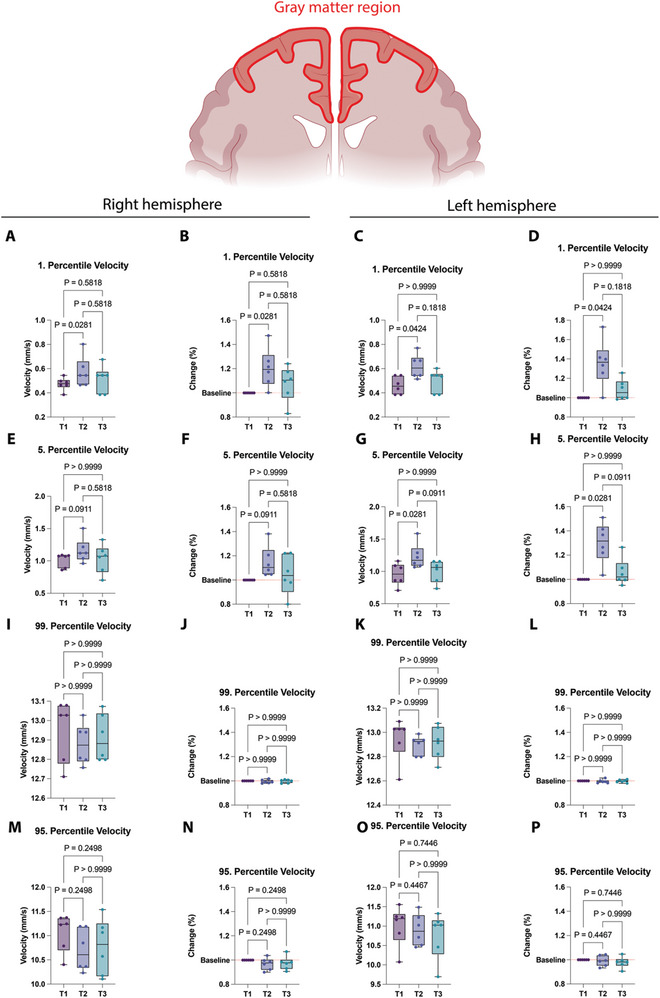
Group‐level flow changes during therapy. A) First percentile of absolute microbubble flow velocities in the left cortical region (T1: *n* = 6, T2: *n* = 6, T3: *n* = 6). B) Relative changes of first percentile of microbubble flow velocities with respect to baseline (T1 = 100%) in the left cortical region (T1: *n* = 6, T2: *n* = 6, T3: *n* = 6). C) First percentile of absolute microbubble flow velocities in the right cortical region (T1: *n* = 6, T2: *n* = 6, T3: *n* = 6). D) Relative changes of 1^st^ percentile of microbubble flow velocities with respect to baseline (T1 = 100%) in the right cortical region (T1: *n* = 6, T2: *n* = 6, T3: *n* = 6). (E) 5th percentile of absolute microbubble flow velocities in the left cortical region (T1: *n* = 6, T2: *n* = 6, T3: *n* 6). F) Relative changes of 5th percentile of microbubble flow velocities with respect to baseline (T1 = 100%) in the left cortical region (T1: *n* = 6, T2: *n* = 6, T3: *n* = 6). (G) 5th percentile of absolute microbubble flow velocities in the right cortical region (T1: *n* = 6, T2: *n* = 6, T3: *n* = 6). (H) Relative changes of 5th percentile of microbubble flow velocities with respect to baseline (T1 = 100%) in the right cortical region (T1: *n* = 6, T2: *n* = 6, T3: *n* = 6). (I) 99th percentile of absolute microbubble flow velocities in the left cortical region (T1: *n* = 6, T2: *n* = 6, T3: *n* = 6). (J) Relative changes of 99th percentile of microbubble flow velocities with respect to baseline (T1 = 100%) in the left cortical region (T1: *n* = 6, T2: *n* = 6, T3: *n* = 6). K) 99th percentile of absolute microbubble flow velocities in the right cortical region (T1: *n* = 6, T2: *n* = 6, T3: *n* = 6). L) Relative changes of 99^th^ percentile of microbubble flow velocities with respect to baseline (T1 = 100%) in the right cortical region (T1: *n* = 6, T2: *n* = 6, T3: *n* = 6). M) 95th percentile of absolute microbubble flow velocities in the left cortical region (T1: *n* = 6, T2: *n* = 6, T3: *n* = 6). N) Relative changes of 95th percentile of microbubble flow velocities with respect to baseline (T1 = 100%) in the left cortical region (T1: *n* = 6, T2: *n* = 6, T3: *n* = 6). O) 95th percentile of absolute microbubble flow velocities in the right cortical region (T1: *n* = 6, T2: *n* = 6, T3: *n* = 6). (P) Relative changes of 95th percentile of microbubble flow velocities with respect to baseline (T1 = 100%) in the right cortical region (T1: *n* = 6, T2: *n* = 6, T3: *n* = 6). Dots show individual patient values. Parameters tested with non‐parametric Friedman test and Dunn's post‐test. Created with BioRender.com.

To quantify the group effects more precisely, we looked at the flow velocity changes for each individual patient. This was done by calculating the 1st and 5th percentiles and the 99th and 95th percentiles, which represent very slow and very fast‐flowing structures respectively. For these analyses, the patient with large infarcted regions and no T3 timepoint was excluded. We were able to attribute these effects to each hemisphere separately and recognized a pronounced flow acceleration of very slow structures (1^st^ percentiles) in both hemispheres (**Figure** **4**A–D). These effects were also detectable for the 5th percentile of flow velocities (Figure 4E–H). In contrast, the faster vessels (95th and 99th percentiles) were not affected by flow changes during therapy (Figure 4I–P).

### 2.6 Deciphering Region‐Specific Reorganization in Macro‐ and Microvascular Spatiotemporal Resolution

**Figure 5:**
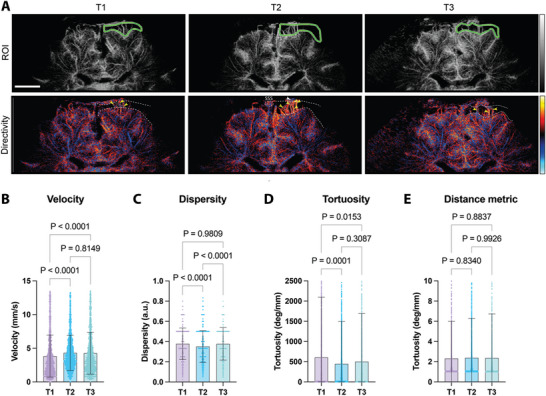
CEUS during cortical remodulation: Post‐interventional activity beyond cortical vascularity. A) Ultrasound localization microscopy density (upper) and directivity maps (lower row) for all investigated timepoints (T1–T3, left to right) and schematic outline of the subarachnoid space (green region of interest). Yellow arrows mark individual vessels, white arrows point to the medullary space, and SSS = superior sagittal sinus. Bar represents 1cm. B) Microbubble flow velocities (T1: *n* = 2123, T2: *n* = 1878, T3: *n* = 2124), C) dispersity (T1: *n* = 1397, T2: *n* = 1519, T3: *n* = 1531), D) tortuosity (T1: *n* = 2204, T2: *n* = 1995, T3: *n* = 2264), and E) distance metric (T1: *n* = 2208, T2: *n* = 1996, T3: *n* = 2265) for all time points in one patient. Parameters tested with one‐way ANOVA with Tukey post‐test. Bars represent SD. Created with BioRender.com.

Microstructural analysis with ULM in the same cortical regions revealed activating processes beyond cerebral vascularity. In contrast to most experiments on animal models, the anatomy of the brain – in particular the skull and bones – is completely preserved in this clinical study as we used the fontanelle as an imaging window. This enabled us to analyze the subarachnoid vascular structures during the therapeutic interventions. In this context, we found the activation of cortico‐medullary connections and bridging veins after interventional occlusion of pathological arteriovenous shunt connections (**Figure** **5**A). We can show that singular microbubbles being initially tracked at the cortex go beyond the level of the venous sinus or drainage area and reach the cranial bone (marrow) (Videos S1–S3, Supporting Information). As compared to baseline, the individual microbubble flow was increased from 3.8 ± 3.1 mm s^−1^ at T1 to 4.3 ± 2.6 mm s^−1^ at T2 (*p* < 0.0001) and remained increased to 4.3 ± 3.1 mm s^−1^ at T3 (*p* < 0.0001) (Figure 5B). These observations can act as a sign of facilitated and increased venous outflow, but possibly also as part of an inflammatory process after embolization. Such extra‐cortical vascular connections were detected more frequently and with an altered velocity distribution after the intervention (T2), however, they were still detectable one week after intervention (T3). To a lesser extent also dispersity (Figure 5C) and tortuosity (Figure 5D) showed temporary alterations after therapeutic intervention. Overall, in principle, the anatomy of the vessels remained unchanged, as indicated by a constant distance metric (Figure 5E).

### 2.7 Microstructural Dynamics as Novel Discriminators of Cerebral Pathology

**Figure 6:**
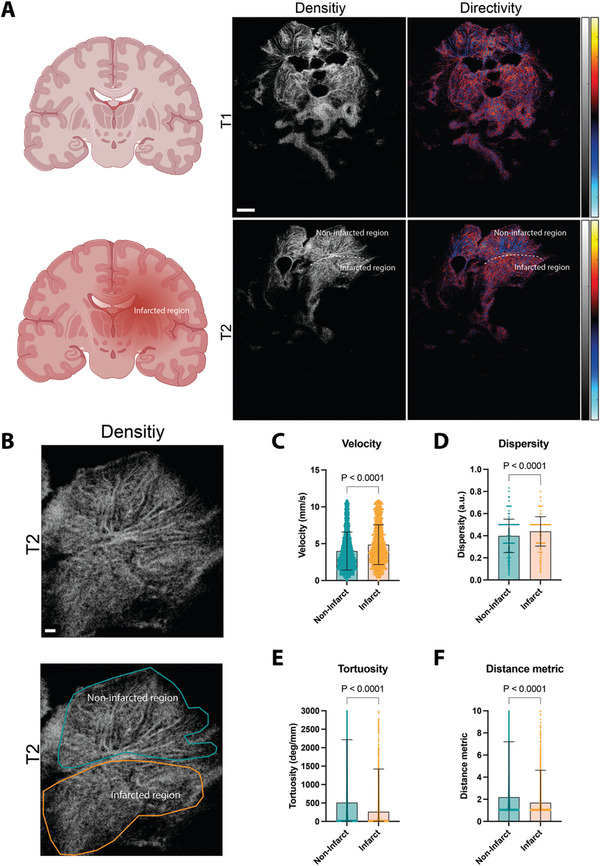
Microvascular dynamics as novel discriminators of cerebral pathology. A) Schematic illustration of cerebral infarction after the treatment (T1 and T2), ultrasound localization microscopy density, and directivity maps (left to right). Blue codes for upward and red for downward localization. Bar represents 1cm. B) Outline of the regions of interest, green = non‐infarcted, viable tissue, orange = infarcted tissue. Bar represents 1 cm. C) Microbubble flow velocities (non‐infarcted: *n* = 11 116, infarcted: *n* = 3665), D) dispersity (non‐infarcted: *n* = 6782, infarcted: *n* = 2085), E) tortuosity (non‐infarcted: *n* = 11 160, infarcted: *n* = 3700), and F) distance metric (non‐infarcted: *n* = 11 169, infarcted: *n* = 3703) for T1 and T2. Bars represent SD. SSS = superior sagittal sinus. Created with BioRender.com.

Due to the severity of the disease, it is difficult to rule out possible complications during such procedures. In one patient, we observed a space‐occupying parenchymal hemorrhage in the left parieto‐occipital hemisphere surrounded by an infarcted area as a postinterventional complication (**Figure** **6**A). By segmenting the infarcted parenchyma from the corresponding viable tissue, we were able to derive individual microbubble flow velocities (Figure 6B). We found a relative increase of microbubble flow velocities in the infarcted area (4.0 ± 2.6 mm s^−1^ vs 4.8 ± 2.7 mm s^−1^, *p* < 0.0001) (Figure 6C). Furthermore, dispersity increased (*p* < 0.0001) (Figure 6D) while tortuosity (*p* < 0.0001) (Figure 6E) and the distance metric decreased (*p* < 0.0001) (Figure 6F). This shows a loss of small microvasculature resulting from cerebral infraction, introducing microstructural analysis with ULM as a novel assessment tool for pathological vascular dynamics.

## 3 Discussion

Since Dussik started his research into a technology he later called “hyperphonography” around the year 1937,^[^ ^17^ ^]^ ultrasound imaging has undergone a significant development making it now available in various shapes and sizes for wide‐spread medical applications.^[^ ^18^ ^]^ In this study, we were able to image the dynamic and ultrastructural reorganization of cortical blood flow after neurovascular interventions using standard clinical hardware. We demonstrate that embolization of AV malformations creates complex and dynamic adaptions of the cerebral blood flow – especially in cortical regions. Moreover, changes in hemodynamics are accompanied by distinct morphological alterations, which can be monitored by tracking individual microbubbles.^[^ ^19^ ^]^ While dynamic, software‐based quantifications of T‐CEUS provide summation signals of signal intensities over time,^[^ ^6^, ^20^ ^]^ ULM allowed qualitative and quantitative microvascular imaging.

VGAM affects cerebral perfusion at various levels: massively reduced arterial resistance leads to redistribution of blood flow in the brain parenchyma, increased venous pressure, and venous congestion.^[^ ^21^ ^]^ Patients with VGAM often present with high‐output heart failure, which may require urgent endovascular treatment for stabilization.^[^ ^22^ ^]^ While vascular malformations are morphologically variable, so are their clinical courses, severity, and treatment approaches. While clinical scoring provides limited prognostic outcome measures,^[^ ^11b^ ^]^ imaging approaches such as MRI or echocardiographic parameters may more properly reflect the course of the disease.^[^ ^23^ ^]^ In this study, we were able to image the changes in the cerebral microvasculature and found that it contributed to the disease pathology and reflected treatment interventions. Despite the clear changes found in the microcirculation of the brain, it remains to be determined what to what degree imaging will correlate to the long‐term functional development and recovery of the patients.

Using clinical standard hardware, ULM density maps provided pixel sizes of 10 µm and allowed precise analysis of grey and white matter structures. First studies now have shown its translation to image organs of adults.^[^ ^19^, ^24^ ^]^ In case of cerebral imaging, most preclinical studies required removal of the bone (craniotomies) to create sufficient imaging windows,^[^ ^25^ ^]^ while we were able to use the fontanelle as an imaging window.^[^ ^26^ ^]^ In the future, volumetric imaging approaches could largely increase the value of ULM by providing information on the entire neonatal microvasculature allowing for enhanced spatial resolution and motion correction in all directions.^[^ ^27^ ^]^ By this, a resolution up to 20–31 µm was already achieved through the intact skull in mice^[^ ^28^ ^]^ and on large animals after craniotomy.^[^ ^29^ ^]^ Similarly, a study in non‐human primates showed feasibility in a depth of 3 cm using an 8‐MHz probe, while a 3‐MHz probe achieved a resolution of 60 𝜇m through the intact skull.^[^ ^30^ ^]^

However, the horizon opened by this ULM goes even further. As already described in experimental studies and dissection preparations, special vascular connections exist in the brain.^[^ ^31^ ^]^ While these microscopic findings in mice point to the fact that there is a direct local interaction between the brain and the skull bone marrow through the meninges,^[^ ^31a^ ^]^ the detection of these skull‐meninges connections (SMC) defies conventional imaging techniques in the clinic. We could observe that occlusion of cerebral vasculature during therapy may also alter the flow of cortico‐medullary as well as bridging veins as a sign of easier and increased venous outflow, but possibly also as part of the inflammatory process following embolization. This potentially represents a target for new therapeutic interventions, as this interface represents a special immunologically active compartment with a unique molecular signature.^[^ ^32^ ^]^ Whereas in the animal model, by creating a boneless window,^[^ ^28^, ^33^ ^]^ these connections are arguably not visible at all, in the clinical context they become detectable by ULM. However, their role in neonates still needs to be elucidated.

This study had limitations. First, the number of patients was limited, partly due to the rarity of the disease. While clinical standard hardware was used in this study, more specific and dedicated instrumentation may achieve significantly better imaging results. On the other hand, the principles shown here are universally applicable to existing technology and can be applied to other diseases.

The results of our study demonstrate the highly effective combination of CEUS and ULM and show that super‐resolution imaging is clinically translatable and enables non‐invasive microstructural and functional vascular imaging in human neonates at unprecedented spatiotemporal resolution.

## 4 Experimental Section

### Study Design and Patients

This study examined male and female neonates and sex was not considered as a biologic variable. For this prospective single‐center, cross‐sectional diagnostic trial ethical and regulatory approval was obtained from the Ethics Committee of the North Rhine Medical Association, Duesseldorf, Germany (Reference number: 2 021 435). All parents or guardians signed informed consent and the trial was registered (drks.de Identifier: DRKS00030052). Consecutive patients were recruited from July 2022 to February 2023 prior to neurovascular interventions. Participation in the study was optional for the families and not a condition of treatment, nor did it have any influence on it. For this study, a subset of the first *n* = 7 consecutive patients was analyzed. This cohort was recruited independently of previous studies and data have not been included in other reports. For comparison, a cohort of *n* = 8 patients with no cerebral vascular malformations was included, as described earlier (clinialtrial.org ID NCT03215628).^[^ ^6c^ ^]^

### Clinical Data and Standard Assessments

All patients received continuous medical monitoring according to standard operating procedures during hospitalization in a dedicated neonatal intensive care unit, during ultrasound examinations, and during embolization. Individual patient data were recorded, including gestational age, postnatal age, weight, sex, clinical scores (Bicêtre neonatal evaluation score,^[^ ^11b^ ^]^ neonatal multiple organ dysfunction score),^[^ ^14a^ ^]^ oxygen saturation, oxygen demand, mean arterial pressure, ventilation time, length of stay in the pediatric intensive care unit, and total time of hospitalization. Routine and scientific imaging parameters (ultrasound, echocardiography, MRI, CT) were also assessed. Blood gas analyses and other clinical chemistry parameters (blood count, renal values, liver values, coagulation, NT‐ProBNP) were collected according to routine clinical practice as part of standard monitoring.

### Assessment of Clinical Scores

Disease severity was evaluated using two clinical scoring systems as described previously.^[^ ^23b^ ^]^ Initially, before elective intubation, the Bicêtre neonatal evaluation score was used to establish the maximum clinical impairment prior to cerebral magnetic resonance imaging (cMRI) and angiography (Bicêtre_pre_). After the embolization, the Bicêtre neonatal evaluation score was adjusted by assessing the maximum renal and hepatic dysfunction post‐embolization (Bicêtre_post_). The Bicêtre neonatal evaluation score evaluates five objective clinical parameters, namely cardiac function, respiratory function, cerebral function, renal function, and hepatic function, to produce a score between 0 to 21 points (lower scores indicate higher disease severity).^[^ ^11b^ ^]^ To evaluate the extent of multiorgan dysfunction throughout disease progression, the modified neonatal multiple organ dysfunction (NEOMOD) score was employed.^[^ ^14^ ^]^ The NEOMOD assessment scores eight organ systems, such as the central nervous, hemocoagulation balance, respiratory, gastrointestinal, cardiovascular, renal, acid‐base balance, and microvascular systems. Scoring between 0 and 16 points, it increases in severity with multiorgan dysfunction. The scores were collected prior to planned intubation for initial embolization and daily after embolization during a 7‐day period. NEOMOD_max_ refers to the highest observed value.

### Endovascular Therapy

After ultrasound‐guided puncture of the femoral artery and vein plus positioning 3 French (F) sheaths (IVA3F; BALT, Montmorency, France), a 1.5 F microcatheter (Marathon Flow Directed Microcatheter; Medtronic, Minneapolis, USA) was used for arterial probing and a 1.7 F microcatheter (Echelon 10 Microcatheter; Medtronic, Minneapolis, USA) for venous probing of the arteriovenous shunts. A combined therapy with both arterial and venous approaches for precise occlusion of high‐flow arteriovenous fistulas accurately at the shunting point with coils and/or ethylene vinyl alcohol (Onyx, Medtronic, Irvine, CA, USA) was used for embolization. Superselective arterial feeder probing in combination with a retrograde transvenous approach using the “looping technique” or “kissing microcatheter technique” was performed to gain direct access to the inflowing artery at the entry point to the dilated persistent median prosencephalic vein of Markowski.^[^ ^34^ ^]^

### Ultrasound Imaging Procedures

Transfontanellar CEUS (T‐CEUS) imaging was performed as described in previous studies.^[^ ^6^ ^]^ Briefly, for all investigations, a high‐end ultrasound system (GE Logiq E10s R3, GE Healthcare, Boston MA, USA) together with a micro curved array probe (C3‐10, GE Healthcare, Boston MA, USA) was used to image with a low mechanical index preset (MI: 0.07), a frequency of 4 MHz and a frame rate of 14 Hz. All investigations were performed by an ultrasound‐trained (German Association for Ultrasound in Medicine, Level II) and board‐certified neonatologist (S.S.). First, standardized cerebral sonography was performed to identify focal abnormalities in the cortex, grey matter, or basal ganglia. Then, the probe was positioned directly at the great fontanelle and maintained constant in a coronal plane just beyond the foramen of Monroi adjusted to section at the level of the island region during the entire acquisition.

Intravenous microbubbles (SonoVue, Bracco, Italy) were administered through a central venous catheter (femoral vein, brachiocephalic vein) as a bolus of 0.08 mL kg^−1^ and flushed with 3 mL NaCl 0.9%. Then, a (DICOM) video clip of at ≈80–120 s in the middle coronary section was recorded. A second scan was obtained if the image quality was insufficient, e.g., due to movement artifacts or focal abnormalities causing image disturbances. The second scan was only performed after contrast agent had been completely washed out and no residues were in the vascular access. For comparison, *n* = 8 patients with coarctation of aorta (and without cerebral vascular malformation) undergoing neonatal heart surgery served as controls.^[^ ^6c^ ^]^ In consideration of ethical constraints, it was not feasible to incorporate a healthy control group.

### Imaging Time Points

Depending on the individual health status while maintaining normal values for potential confounding factors (mean arterial blood pressure, arterial pCO_2_, arterial pO_2_) of the subject, imaging was scheduled within 24 h before endovascular therapy (T1), within 24 h after endovascular therapy (T2), and one week after therapy (T3).

### Software‐Based Analysis and Time‐Intensity‐Curve Quantification

One independent investigator, not involved in patient care or scanning of the subjects, performed time‐intensity curve (TIC) quantifications. For this, all video clips were exported in DICOM format and quantification was performed using dedicated commercial software (VueBox, Version 7.1.5.58024, Bracco Suisse SA, Plan‐lesouates, Switzerland). As described in detail earlier, this approach enables to generate quantitative parameters as surrogates of microbubbles flow over time in a predefined region of interest (ROI).^[^ ^6^ ^]^

For this study, parameters were measured bilaterally in the hemispheres and three predefined ROIs (cortical regions, white matter regions, putamen). The software generates eleven dynamic flow parameters: peak enhancement (PE), wash‐in area under the curve (WIAUC), wash‐in perfusion index (WiPI), wash‐in rate (WIR), wash‐out area under the curve (WoAUC), wash‐in and wash‐out area under the curve (WiWoAUC), wash‐out rate (WoR), time to peak (TTP), rise time (RT), mean transit time (local) (mTTl) and fall time (FT). If single parameters could not be calculated, these data points were excluded from the final analysis.

### Ultrasound Localization Microscopy (ULM)

The codes that allowed the reconstruction of vascular mapping by Ultrasound Localization Microscopy (ULM) have previously been published^[^ ^24b,e^ ^]^ and are available in the following GitHub repository: https://github.com/AChavignon/PALA. First, T‐CEUS imaging clips were split into blocks of 200 frames each. Then, a spatiotemporal filter (SVD) with a threshold of 5/200 eigenvalues to enhance the microbubble signal was applied. Then it was localized the center of microbubbles with the radial symmetry method and tracked them with the simple tracker toolbox.^[^ ^24b^ ^]^ This tracking step was performed using a maximum distance between microbubbles ≈2 λ and a minimum track duration of 1 frame. By interpolating and accumulating all the tracks, a microvascular ULM density map with a final grid with 10 times higher resolution compared to the original one was reconstructed, i.e., a final pixel size of 10 µm. ULM directivity maps were also reconstructed by projecting the direction of the tracks, encoded in red if the track was approaching the probe, and blue if it was moving away from it. All these post‐processing steps were performed with Matlab R2020a. They can also be realized with the stand‐alone applications LOTUS and AKEBIA, without the need for a Matlab license.^[^ ^24b,e^ ^]^

### ULM Metrics Quantification

To compare ULM maps in the different brain regions, it was manually segmented the grey and white matter regions in each scan. The segmentation of the brain regions was carried out by an ultrasound‐certified pediatrician/neonatologist and confirmed by another. The analysis was carried out by a third person. Each in one example patient, an infarcted region and not infarcted region (Patient 5) and the subarachnoid vasculature (Patient 1) for all time points were segmented. In each of these areas and for each of these time points, individual values were calculated for:

‐ The number of localizations that corresponds to the number of localized microbubbles in each area in arbitrary units (a.u.): the number of microbubbles within an area is a parameter reflecting blood density. The more blood volume is present in an area, the more localizations, and therefore MBs will be recognized. The parameter is useful for calculating (re)perfusion of a specific brain area, which has also been shown recently to assess perinatal stroke.^[^ ^26^ ^]^
‐ The number of upward localizations that correspond to the microbubbles that are directed toward the probe (encoded in red in the ULM directivity maps) in arbitrary units (a.u.).
‐ The number of downward localizations corresponding to microbubbles directed away from the probe (coded blue in the ULM directivity maps) in arbitrary units (a.u.). The number of upward and downward localizations serve as parameters in addition to conventional Doppler analysis since blood flow directivity is visualized. Superior to ultrasound Doppler analysis directivity can be captured in much greater spatiotemporal resolution and vessels up to micron scale.
‐ The overall length of tracks that corresponds to the number of localizations constituting a track in arbitrary units (a.u.).
‐ The velocity consists of dividing the distance covered by the track by the time it takes the track to cover that distance in millimeters per second (mm s^−1^).
‐ The dispersity is defined as the number of times that a track changed direction (>20° change) divided by the number of points inside the track in arbitrary units (a.u.).
‐ The tortuosity is defined as the sum of the angles covered by the track, divided by the distance between the first point and the last point of the track^[^ ^35^ ^]^ in degree per millimeter (deg mm^−1^).
‐ The distance metric (DM) corresponds to the cumulative distance traveled by the track divided by the distance between the first and the last point of this track^[^ ^35^ ^]^ as a ratio without a unit.

### Magnetic Resonance Imaging and Scoring

Magnetic resonance imaging (MRI) examinations were performed according to the following protocol, as previously described.^[^ ^36^ ^]^ Briefly, the following imaging data were acquired using a 1.5 T scanner (Magnetom Aera, Siemens, Erlangen, Germany) under general anesthesia without intravenous gadolinium administration: coronal T2 inversion recovery sequence, axial T2 weighted images turbo spin echo sequences, transversal T2 weighted turbo spin echo sequences with 5 mm slice thickness, sagittal T2 weighted images turbo spin echo sequences with 3 mm slice thickness, time‐of‐flight magnetic resonance angiography (TOF MRA), axial T1 weighted images spin echo sequences, axial diffusion‐weighted imaging, and fat‐saturated T2 weighted images turbo spin echo sequences without flow compensation with a slice thickness of 2 mm.

The pre‐and post‐treatment cerebral magnetic resonance imaging (cMRI) was assessed by an experienced neuroradiologist (NRD), who was blinded to the clinical course and management. As described previously, a standardized scoring system for structural brain abnormalities was used.^[^ ^15^, ^23^ ^]^ Pre (MRI(WM)_pre_) and post‐treatment white matter abnormalities (MRI(WM)_post_) were assessed using a three‐point scale in five anatomical regions for a total white matter score. The WM score consisted of four grades: normal (5–6 points), mildly abnormal (7–9), moderately abnormal (10–12), and severely abnormal (13–15). Pre (MRI(GM)_pre_) and post‐treatment grey matter abnormalities (MRI(GM)_post_) were assessed using a three‐point scale in three regions. A total GM score was calculated by summing up the points from each region and graded into normal (3–5 points) and abnormal (6–9). Using GM and WM subscores, a total score pre‐ (MRI(total)_pre_) and post‐treatment (MRI(total)_post_) were calculated. A total score of 19–24 points was considered as severe brain injury.

### Statistical Analysis

Data are given as mean ± SD or individual data points. Statistics were performed with GraphPad Prism (Version 10.1.0, GraphPad, LaJolla, CA, USA). Differences in CEUS parameters in relation to baseline were tested with the Kruskal‐Wallis test and Dunn's post‐test for correction of multiple testing. For individual ULM parameters, an unpaired two‐sided parametric *t*‐test, or in the case of multiple groups, a one‐way ANOVA with Tukey post‐test was used. Correlation maps are given with Spearman's correlation coefficient (R_s_). *p*‐values <0.05 were considered statistically significant.

## Conflict of Interest

APR and FK are co‐inventors on an EU patent (EP 19 163 304.9). FK is a member of the advisory board of iThera Medical GmbH. APR and FK received travel support from iThera Medical GmbH, Germany. APR and FK report lecture fees from Sanofi Genzyme. FK reports lecture fees from Siemens Healthcare GmbH. OC is a co‐inventor of an ultrasound super‐resolution patent (PCT)/FR2011/052810. OC is also a co‐founder of ResolveStroke. FB is a shareholder of the company Bentley InnoMed GmbH.

## Author Contributions

H.M. and F.K. contributed equally to this work. S.S. conceptualized, designed and obtained approval the clinical study; S.S. performed ultrasound imaging investigations; J.J., H.R., and N.B. provided support on the methodology; F.B.N. was involved in the clinical care of the patients; N.R.D., M.S.H., and F.B. were involved in the therapeutic interventions and magnetic resonance imaging on patients; L.D. and O.C. provided analyses codes; L.D., E.N., A.B., V.D., A.P.R., O.C., and H.M. conducted the analyses on imaging data; S.S., H.M., and F. K. supervised data analyses, produced the figures and composed the manuscript; A.H., U.F.M., J.W., and C.D.S. provided valuable discussion on the study results and edited on the manuscript. All authors contributed to writing the manuscript and approved the final version.

## Supporting information



Supporting Information

Supplemental Video 1

Supplemental Video 2

Supplemental Video 3

## Data Availability

The data that support the findings of this study are available on request from the corresponding author. The data are not publicly available due to privacy or ethical restrictions.
