# Supplementary material for: Ultrasound Super‐Resolution Imaging of Neonatal Cerebral Vascular Reorganization
Source: Adv Sci (Weinh). 2025 Feb 3;12(12):2415235. doi: 10.1002/advs.202415235 (PMC11948062; doi:10.1002/advs.202415235)
Supplement: Supplementary file 1 — Supporting Information [file ADVS-12-2415235-s003.docx]

Supporting Information

Ultrasound super-resolution imaging of neonatal cerebral vascular reorganization

*Simone Schwarz, Louise Denis, Emmanuel Nedoschill, Adrian Bühler, Vera Danko, Alina Hilger, Francisco Brevis Nuñez, Nikola Reinhard Dürr, Martin Schlunz-Hendann, Friedhelm Brassel, Ursula Felderhoff-Müser, Heiko Reutter, Joachim Woelfle, Jörg Jüngert, Christian Dohna-Schwake, Nora Bruns, Adrian P. Regensburger, Olivier Couture, Henriette Mandelbaum and Ferdinand Knieling**

# Supplementary Figures

**
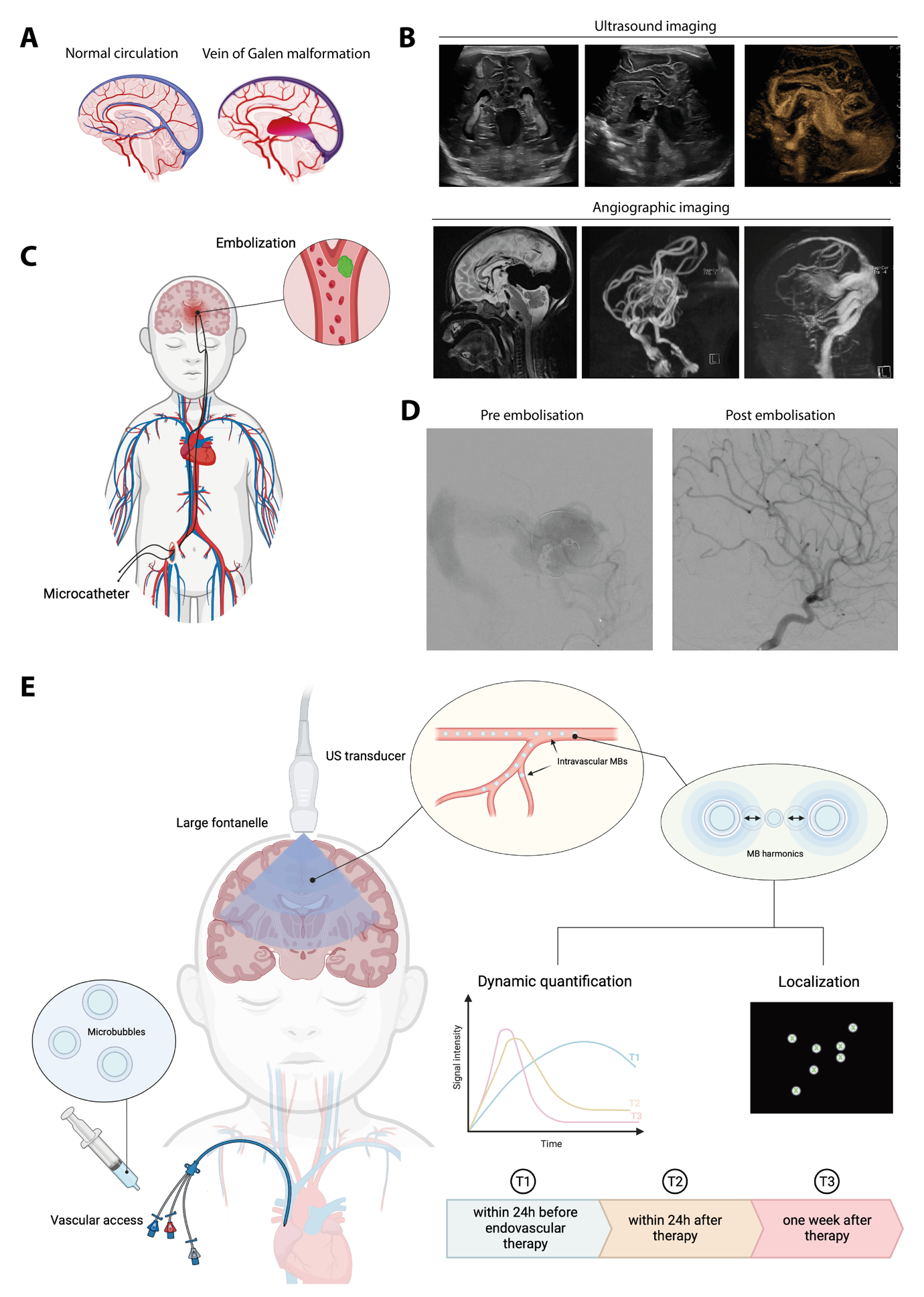
**

**Figure S1.** Schematical description of the disease pathology and the study flow. (**A**) Schematic illustration of a Vein of Galen (aneurysmal) malformation with pathologic cerebral arteriovenous connections. (**B**) Standard clinical ultrasound, contrast-enhanced ultrasound (upper row) and magnetic resonance angiography (lower row), demonstrating the dilated (aneurysmal) malformation and pathologic arteriovenous connections. (**C**) Schematic illustration of therapeutic approach using arterial and venous catheters for superselective embolization of pathologic vasculature. (**D**) Angiographic view of contrast distribution in the anterior stromal region on the right before (left) and after interventional therapy (right). (**E**) Schematic illustration of the proposed imaging approach using ultrasound contrast agents and imaging through the large fontanelle at three different time points during therapy (T1 - T3). The data was processed to generate time-intensity curves of ultrasound contrast agent signals and localization and tracking of individual microbubbles for ultrasound localization microscopy. Created with BioRender.com.

# Supplementary Tables

## Table S1 - Patient characterization and clinical data

| **Patient**  **No.** | **Sex** | **Gestational Age** | **Weight (g)** | **Age at admission**  **(d)** | **VGAM subtype** | **Age at 1^st^ embolization/**  **cMRI**  **(d)** | **No. of interventions during 1^st^ inpatient stay** | **Major Complications** |
| --- | --- | --- | --- | --- | --- | --- | --- | --- |
| 1 | M | 37 1/7 | 3000 | 2 | mural | 5 | 1 | none |
| 2 | M | 38 1/7 | 2980 | 1 | mixed type | 9 | 1 | none |
| 3 | M | 38 0/7 | 3090 | 1 | mural | 2 | 1 | none |
| 4 | M | 37 5/7 | 3360 | 2 | choroidal | 2 | 1 | yes |
| 5 | M | 34 6/7 | 2710 | 1 | choroidal | 2 | 1 | yes |
| 6 | M | 35 5/7 | 2450 | 1 | choroidal | 3 | 2 | yes |
| 7 | M | 40 1/7 | 4600 | 1 | mural | 2 | 1 | none |

F = Female, M = Male, VGAM = Vein of Galen aneurysmal malformation, cMRI = cerebral magnetic resonance imaging

## Table S2 - Clinical scores and MR imaging

| **Patient**  **No.** | **BNES**  **pre** | **BNES**  **post** | **NEOMOD**  **max** | **MRI(WM) pre** | **MRI(GM) pre** | **MRT(total) pre** | **MRI(WM) post** | **MRI(GM) post** | **MRI(total)**  **post** | **Mortality** |
| --- | --- | --- | --- | --- | --- | --- | --- | --- | --- | --- |
| 1 | 10 | 10 | 5 | 6 | 4 | 10 | 7 | 4 | 11 | alive |
| 2 | 21 | 21 | 3 | 6 | 3 | 9 | 7 | 3 | 10 | alive |
| 3 | 16 | 14 | 8 | 9 | 4 | 11 | 5 | 3 | 8 | alive |
| 4 | 11 | 11 | 10 | 7 | 5 | 11 | 13 | 5 | 18 | alive |
| 5 | 10 | 5 | 12 | 10 | 5 | 15 | 12 | 6 | 18 | died |
| 6 | 10 | 9 | 9 | 7 | 3 | 10 | 13 | 6 | 19 | died |
| 7 | 21 | 21 | 2 | 6 | 3 | 9 |  |  |  | alive |

BNES = Bicetrê neonatal evaluation score, NEOMOD = neonatal multiple organ dysfunction score, WM = white matter, GM = grey matter, cMRI = cerebral magnetic resonance imaging

# Supplementary Movies

## Movie S1. Contrast-enhanced ultrasound video sequences for all timepoints (T1 – T3) of an exemplary patient. Videos show a coronal section at a middle coronal plane.

**Movie S2.** Individual microbubble tracking. The upper row shows directivity maps of the entire brain at all time points (T1 – T3) with schematic outline of the subarachnoid space (dotted lines). Yellow arrows mark individual vessels, SSS = superior sagittal sinus. The lower row shows color-coded tracks of individual microbubbles (red dots) at all time points (T1 – T3) of the left cortex area.

**Movie S3.** Individual microbubble tracking of the section of the left cortex area at time T3. The video shows color-coded tracks of individual microbubbles (red dots).
